# In-Vitro Characterization of Growth Inhibition against the Gut Pathogen of Potentially Probiotic Lactic Acid Bacteria Strains Isolated from Fermented Products

**DOI:** 10.3390/microorganisms9102141

**Published:** 2021-10-13

**Authors:** Ji Young Jung, Sang-Soo Han, Z-Hun Kim, Myung Hoo Kim, Hye Kyeong Kang, Hyun Mi Jin, Mi Hwa Lee

**Affiliations:** 1 Microbial Research Department, Nakdonggang National Institute of Biological Resources (NNIBR), 137, Donam 2-gil, Sangju-si 37242, Gyeongsangbuk-do, Korea; sshan0324@add.re.kr (S.-S.H.); kimzhun@nnibr.re.kr (Z.-H.K.); khk7018@nnibr.re.kr (H.K.K.); hmjin@nnibr.re.kr (H.M.J.); blume96@nnibr.re.kr (M.H.L.); 2 Department of Animal Science, College of Natural Resources & Life Science, Pusan National University, Miryang-si 50463, Gyeongsangnam-do, Korea; mhkim18@pusan.ac.kr

**Keywords:** Lactic acid bacteria (LAB), gut microbiota, pathogen growth inhibition, in vitro investigation

## Abstract

Lactic acid bacteria (LAB) are probiotic candidates that may restore the balance of microbiota populations in intestinal microbial ecosystems by controlling pathogens and thereby promoting host health. The goal of this study was to isolate potential probiotic LAB strains and characterize their antimicrobial abilities against pathogens in intestinal microbiota. Among 54 LAB strains isolated from fermented products, five LAB strains (NSMJ15, NSMJ16, NSMJ23, NSMJ42, and NFFJ04) were selected as potential probiotic candidates based on in vitro assays of acid and bile salt tolerance, cell surface hydrophobicity, adhesion to the intestinal epithelium, and antagonistic activity. Phylogenetic analysis based on 16S rRNA genes showed that they have high similarities of 99.58–100% to *Lacticaseibacillus* *paracasei* strains NSMJ15 and NFFJ04, *Lentilactobacillus parabuchneri* NSMJ16, *Levilactobacillus* *brevis* NSMJ23, and *Schleiferilactobacillus harbinensis* NSMJ42. To characterize their antimicrobial abilities against pathogens in intestinal microbiota, the impact of cell-free supernatant (CFS) treatment in 10% (*v*/*v*) fecal suspensions prepared using pooled cattle feces was investigated using in vitro batch cultures. Bacterial community analysis using rRNA amplicon sequencing for control and CFS-treated fecal samples at 8 and 16 h incubation showed the compositional change after CFS treatment for all five LAB strains. The changed compositions were similar among them, but there were few variable increases or decreases in some bacterial groups. Interestingly, as major genera that could exhibit pathogenicity and antibiotic resistance, the members of *Bacillus*, *Escherichia*, *Leclercia*, *Morganella*, and *Vagococcus* were decreased at 16 h in all CFS-treated samples. Species-level classification suggested that the five LAB strains are antagonistic to gut pathogens. This study showed the probiotic potential of the five selected LAB strains; in particular, their antimicrobial properties against pathogens present in the intestinal microbiota. These strains would therefore seem to play an important role in modulating the intestinal microbiome of the host.

## 1. Introduction

The relationship between intestinal microbiota and host health is being increasingly recognized. It is well established that a healthy intestinal microbiota plays a role in the digestion of nutrients, energy metabolism, protection of bacterial disease, and the development of a proper immune system conducing to host health [1,2,3]. In particular, a well-balanced microbiota can provide colonization resistance against harmful intestinal microbiota members (pathogens) which cause an imbalance in microbiota composition (dysbiosis) and can have other adverse effects, such as bacterial infection, gut inflammation, and disruption of the normal immune system [3,4,5,6].

Controlling pathogenic microorganisms in the intestinal microbial ecosystem is important for maintaining health for humans and animals. Fecal microbiota transplantation (FMT) has recently been applied to directly change GI tract microbiome dysbiosis from pathogen blooms to normalized microbiomes [7,8]. Several alternatives to conventional antibiotics have been recently studied as therapeutic agents to combat pathogens, such as bacteriophages and bacteria-derived antimicrobial peptides (AMPs) [9,10,11]. Probiotics are being used widely as one of the key solutions to restore the balance of the gut microbial ecosystem by controlling pathogens and promoting beneficial functions of the gut, resulting in the amelioration or prevention of intestinal or systemic disease phenotypes [12,13,14].

Lactic acid bacteria (LAB) have a wide range of habitats, including GI tracts, oral tracts, and the vaginal tracts of humans and animals, as well as fermented foods and silages. Belonging to a great variety of genera, they can produce lactic acid and metabolites, including organic acids and antimicrobial compounds [15]. LABs are classified as Generally Recognized As Safe (GRAS) and are being extensively studied to develop probiotic microorganisms for human and animal health by restoring the balance of the intestinal microbial ecosystem, controlling pathogens, and providing beneficial functions, for instance, by means of immunomodulatory effects and anti-inflammatory activities [16,17,18]. In terms of the maintenance and improvement of intestinal microbial balance, many studies have primarily focused on the antagonistic effects of LAB isolates against pathogens as important criteria for selecting probiotics [19,20,21]. In animal science, as alternatives to antibiotics, potential probiotic LABs are being isolated and characterized with a focus on functionalities regarding the control of infectious pathogens [18,22,23].

The main objective of this study was to isolate new potential probiotic LABs and characterize their growth inhibitory effects against gut pathogenic bacteria with a focus on the role of probiotics in the control of pathogenic populations in intestinal microbiota. Molecular approaches, such as high-throughput sequencing (HTS), have led to a more comprehensive understanding of the complex intestinal microbiota compared to traditional microbiological methods [24]. To achieve the objective, we performed probiotic characterization assays using LAB strains isolated from fermented products and assessed bacterial population changes in fecal microbiota by treatment with LAB strains using an in vitro batch culture model and high-throughput amplicon sequencing.

## 2. Materials and Methods

### 2.1. Collection of Samples and LAB Strains

To isolate LAB strains, home-made kimchi, makgeolli, and fermented feed were collected from Gyeongsangbuk-do province, South Korea. Each sample was resuspended with phosphate-buffered saline (PBS, pH 7.4). The mixture was then 10-fold serially diluted in PBS (pH 7.4). Diluents were then spread onto MRS agar (Difco, Detroit, MI, USA) plates and the plates were then incubated at 30 °C for 3 days. After incubation, colonies with “LAB-like” morphology were picked at random and directly resuspended in MRS broth (Difco). After incubation at 30 °C for 2 days, the cells were stored in 20% (*v*/*v*) glycerol as stock solutions at −70 °C. These bacterial cells were used to screen LABs harboring probiotic potentials.

### 2.2. Selection and Characterization of Potentially Probiotic LAB Strains Using In-Vitro Assays

#### 2.2.1. Acid Tolerance Test

Primary selection of potential probiotic bacteria was done by testing isolates for their high acid-tolerance in low pH conditions. The acid resistance of bacterial isolates was assayed using the method of Wei et al. [25]. Briefly, bacterial isolates were subcultured in MRS broth and washed twice with PBS (pH 7.4). Bacterial suspension (OD_600_ = 1.0) (1 mL) in PBS (pH 7.4) was centrifuged at 13,000× *g* for 10 min and the pellet was suspended in 1 mL of PBS (adjusted to pH 2.5 with HCl) to simulate the low pH environment of the stomach. The suspension was incubated at 37 °C for 2 h. Viable cells were enumerated on MRS agar with a standard plate counting method after 0 and 2 h of incubation, respectively. The survival rate (%) was calculated as the number of viable cells (CFU/mL) compared to that at 0 h of incubation and all bacterial strains exhibiting good acid-tolerance were subjected to assays of bile salt tolerance, cell-surface hydrophobicity, and bacterial adhesion for final selection. All measurements were done in triplicate.

#### 2.2.2. Bile Salt Tolerance Test

Bile salt tolerance was determined according to the method described previously [23,26]. Briefly, 1 mL aliquot of bacterial culture (OD_600_ = 1.0) in MRS was centrifuged at 13,000× *g* for 10 min and the pellet was suspended in MRS broth (control) or MRS broth containing 0.3% (*w*/*v*) bile salts (Sigma–Aldrich, St. Louis, MO, USA), respectively. Suspensions were incubated at 37 °C for 3 h. Viable cells were then enumerated on MRS agar with a standard plate counting method. The survival rate (%) in bile salt was calculated based on the number of viable cells (CFU/mL) in the MRS broth (control) after 3 h of incubation.

#### 2.2.3. Evaluation of Cell-Surface Hydrophobicity

Cell-surface hydrophobicity was determined according to the method described previously [23,27]. Briefly, 1 mL of bacterial cell suspension (OD_560_ = 1.0 (Ab_0_)) in PBS (pH 7.4) was added with 0.2 mL of n-hexadecane (Sigma–Aldrich) and mixed vigorously for 2 min. After incubation at 37 °C for 1 h, OD_560_ of the aqueous phase was determined (Ab_1_). The percentage of MATS (Microbial Adhesion To Solvents) was calculated using the following equation: %MATS = (Ab_0_ − Ab_1_)/Ab_0_ × 100.

#### 2.2.4. Bacterial Adhesion to Caco-2 Cells

A Caco-2 cell adhesion assay was modified from a previously reported method [28]. Briefly, Caco-2 cells were seeded into 24-well plates in Dulbecco’s Modified Eagle Medium (DMEM) with 1% penicillin-streptomycin (PS) and 20% fetal bovine serum (FBS). When Caco-2 cells were 95–99% confluent, Caco-2 cells were washed with DMEM (without PS and FBS). 0.1 mL bacterial strain (OD_600_ = 1.0) in DMEM was added to the monolayer of Caco-2 cells on the well and incubated in 5% CO_2_–95% air at 37 °C for 1 h. After incubation, DMEM containing non-attached bacteria was removed and the cells were washed twice with DMEM. Then, 0.5 mL of Triton X-100 (1% *v*/*v*) was added and incubated for 5 min to lyse Caco-2 cells. After adding 0.5 mL of 1X PBS (pH 7.4), 10-fold serial dilutions were plated onto MRS agar plates and the attached bacterial cells were counted. The adhesion rate (%) was calculated based on the number of viable cells in the 0.1 mL of bacterial strains (OD_600_ = 1.0) initially added.

#### 2.2.5. Evaluation of Antagonistic Activities

Antagonistic activities were determined according to the double layer agar method [23]. *Escherichia coli* CCARM 1G440 and *Staphylococcus aureus* CCARM 3A860, as two representative Gram-negative and Gram-positive pathogenic bacteria harboring antibiotic resistance, were used as indicator strains. After 1 μL of bacterial culture (OD_600_ = 1.0) in MRS was spotted onto MRS agar plates, the plates were incubated at 30 °C for 48 h. After incubation, the growth was stopped by exposure to chloroform for 20 min [29]. Residual chloroform was allowed to evaporate, and the plates were over-layered with 3.5 mL of low-melt BHI with 2.0% (*w*/*v*) low melting agar (Bio-Rad, Hercules, CA, USA), including indicator strain, to a final concentration of approximately 10^6^ CFU/mL. After the top agar had hardened, plates were incubated at 37 °C for 48 h and the antagonistic activities of bacterial cells were evaluated based on the diameter of the inhibition zones formed around bacterial colonies.

#### 2.2.6. Molecular Identification and Phylogenetic Analysis Based on 16S rRNA Genes

Colonies were resuspended in 100 μL of 5% (*w*/*v*) chelex-100 solution (Bio-Rad) and boiled for 10 min to extract crude genomic DNA. PCR amplification and sequencing of 16S rRNA genes from crude genomic DNAs were performed as described previously [30]. Obtained 16S rRNA gene sequences were blasted against the EzBioCloud prokaryote 16S rRNA gene database [31] to determine similarities with type strains. Obtained 16S rRNA gene sequences of selected bacterial strains and closely related taxa were aligned using ClustalW multiple alignments [32] and a phylogenetic tree was generated using the neighbor-joining (NJ) method available in the MEGA5 software [33].

#### 2.2.7. Phenotypic Characterization

Carbohydrate assimilations of selected LAB strains were identified on API 50 CH (bioMérieux, Marcy l’Etoile, France). NaCl growth range was investigated in MRS broth containing different NaCl concentrations (0–12% at 1% intervals) at 30 °C for 4 days. Acceptable growth temperatures were tested by monitoring their growths at different temperatures (5–50 °C, 5 °C intervals) for 4 days. Acceptable pH values were tested in MRS broth having different pH values (3.0–10.0, 0.5 unit intervals) at 30 °C for 4 days. Anaerobic growth was evaluated on MRS agar under anaerobic conditions (with 4–10% CO_2_) using the GasPak^TM^ systems (BD, Sparks, MD, USA) after 4 days of incubation at 37 °C. Hemolytic activity was determined by streaking onto agar plates containing 5% sheep blood. After incubation at 37 °C for 48 h, hemolytic reactions were examined for signs of β-hemolysis (a clear halo around the colonies), α-hemolysis (a green halo around the colonies), or γ-hemolysis (no halo around the colonies).

### 2.3. In-Vitro Characterization of Growth Inhibitory Activity against the Gut Pathogen of Selected LAB Strains using Microbiota Composition Analysis

#### 2.3.1. In-Vitro Culture Condition of Fecal Microbial Populations and Sampling

To assess the impact of selected LAB strains in terms of gut pathogen inhibition, cattle feces were collected from a livestock farm in Gyeongsangbuk-do province, South Korea. To obtain the fecal microbial populations, including many types of pathogens, fecal samples were pooled and a pooled fecal sample was mixed with PBS (pH 7.4) to a final concentration of 10% (*w*/*v*). Then a low-speed centrifugation (600 *g* for 10 min) was performed to obtain supernatant as a fecal microbiome suspension (FMS). LAB strain-derived cell-free supernatant (CFS) was prepared after sub-culturing for 48 h on MRS broth with inoculation (1%, *v*/*v*) of bacterial cells (OD_600_ = 2.0) grown in MRS broth at 30 °C. To investigate the impact of the CFS on fecal microbial populations, in-vitro batch culture was performed using BHI (Brain Heart Infusion, Difco) broth proven to be effective in cultivating many types of pathogens. Anaerobic BHI broth was made by purging for 30 min with N_2_ to remove O_2_ after autoclaving at 121 °C for 30 min. CFS was treated with 10% (*v*/*v*) to the anaerobic BHI broth with 10% FMS at a final volume of 150 mL using serum bottles in an anaerobic chamber (Coy Vinyl Anaerobic Chamber; Coy Laboratory Products Inc., Grass Lake, MI, USA). Non-CFS-treated samples (serving as a control) contained deionized water instead of CFS. All serum bottles were incubated under anaerobic conditions at 30 °C for 16 h. After 8 mL of the fecal microbiota culture was withdrawn at 8 and 16 h during the incubation, based on the growth curve of the fecal microbiome measured by OD_600_ readings (Figure S4), it was centrifuged at 13,000× *g* for 10 min at 4 °C to harvest microorganisms. Pellets were stored at −80 °C for further analysis.

#### 2.3.2. Illumina MiSeq Paired-End Sequencing

Total DNAs were extracted from all samples using a PowerMax Soil DNA Isolation Kit (MoBio Laboratories, Carlsbad, CA, USA) following the manufacturer’s instructions. The V3-V4 variable region of the 16S rRNA gene was amplified from the extracted DNA using the Illumina 16S metagenomic sequencing library protocol. Two-step PCR reactions were completed. Initially, hypervariable V3 and V4 regions of bacterial 16S rRNA genes were amplified using primers incorporating Illumina overhang adaptors (underlined), 341F (5′-TCGTCGGCAGCGTC-AGATGTGTATAAGAGACAG-CCTACGGGNGGCWGCAG-3′), and 806R (5′-GTCTCGTGGGCTCGG-AGATGTGTATAAGAGACAG-GACTACHVGGGTATCTAATCC-3′). The PCR reaction contained the DNA template (2 ng), forward primer (5 μM), reverse primer (5 μM), Herculase 5X Reaction Buffer, dNTP mixture (25 mM each), 1 U Herculase II polymerase (Agilent Technologies, Santa Clara, CA, USA), and PCR-grade water. PCR amplification was carried out in a T100 thermal cycler (BioRad) using the following parameters: initial denaturation at 95 °C for 3 min, followed by 25 cycles of 95 °C for 30 s, 55 °C for 30 s, and 72 °C for 30 s, then 72 °C for 5 min and held at 4 °C. PCR products were purified using Agencourt AMPure XP Reagents beads (Beckman Coulter, Brea, CA, USA). Purified DNA (5 μL) was used for a second PCR reaction to index each sample, which allowed samples to be pooled for Illumina sequencing on the one flow cell and subsequently demultiplexed for microbial community analysis of each sample. The second PCR reaction contained Illumina Nextera XT index 1 primer (N7xx), Nextera XT index 2 primer (S5xx), Herculase 5X Reaction Buffer, dNTP mixture (25 mM each), 1 U Herculase II polymerase (Agilent Technologies), and PCR-grade water. PCR amplification was completed in a T100 thermal cycler (BioRad) as follows: initial denaturation at 95 °C for 3 min, followed by 10 cycles of 95 °C for 30 s, 55 °C for 30 s, and 72 °C for 30 s, then 72 °C for 5 min and held at 4 °C. PCR products were purified using Agencourt AMPure XP Reagents beads (Beckman Coulter) and pooled in an equimolar fashion. The pooled sample was run on an Agilent Bioanalyzer 2100 (Agilent Technologies) for quality control before sequencing. High-throughput sequencing was performed using a 2 × 300 bp paired-end protocol with an Illumina MiSeq platform (Illumina, San Diego, CA, USA) at the Macrogen sequencing facility (Seoul, South Korea).

#### 2.3.3. Sequencing Processing and Bacterial Community Analysis

After merging paired-end reads by FLASH v1.2.11 [34], pre-processing for de-noising data and OTU clustering at the species level (97% identity cutoff) were performed using CD-HIT-OTU [35]. Taxonomic assignment and diversity statistical analysis were performed with the QIIME2 software pipeline [36]. Representative sequences from each OTU were taxonomically assigned from best-BLAST-hit having at least query coverage (≥85%) and nucleotide identity (≥85%) using BLASTN v2.4.0 based on the NCBI 16S Microbial reference database [37]. A matrix of bacterial relative abundance was built at each taxon level from phylum to species levels. α-diversity was evaluated in QIIME2 by computing Shannon index, Chao1, and Inverse Simpson index at the OTU level. A heatmap was generated to visualize bacterial community changes at the species level from control to tetracycline-treated fecal samples using the heatmap.2 function in the R package “gplots” [38].

## 3. Results

### 3.1. Selection and Characterization of Potential Probiotic Strains

#### 3.1.1. Selection of Five Potential Probiotic LAB Strains

Among a total of 54 bacterial strains isolated from fermented products, the acid-tolerance ability (94.44 ± 4.27%) of NSMJ56 was much higher than LGG (71.60 ± 7.75%) after exposure to pH 2.5 for 2 h. However, another eleven strains (NKJ81, NKJ96, NKJ170, NKJ198, NKJ235, NSMJ15, NSMJ16, NSMJ23, NSMJ27, NSMJ42, and NFFJ04) had similar acid-tolerance abilities to LGG, showing no significant difference among them (Figure S1A). Therefore, these 12 strains were subjected to assays to determine bile salt tolerance, bacterial cell-surface hydrophobicity (% MATS), and bacterial adhesion (%) to Caco-2 cells for secondary selection. After exposure to 0.3% bile salts for 3 h, the survival rates (%) of the 8 strains were similar to that of LGG (89.32 ± 3.72%), except for strains NKJ96, NKJ198, NKJ235, and NSMJ56 (Figure S1B). Among these 12 strains, strains NSMJ15, NSMJ16, NSMJ23, NSMJ42, and NFFJ04 showed much higher MATS values (57.12 to 88.20%) than LGG (26.39%), while another seven strains had much lower MATS values (0.40 to 13.60%) (Figure S2). Adhesion rates (%) to Caco-2 cells of strains NSMJ15, NKJ96, NSMJ23, and NSMJ42 were similar to that of LGG (19.50 ± 7.82%), followed by strains NSMJ16, NSMJ27, NFFJ04, NKJ198, NKJ170, NKJ81, and NKJ235 (Figure S2). Taken together with these results, five strains (NSMJ15, NSMJ16, NSMJ23, NSMJ42, and NFFJ04) were secondarily selected (Table 1) to test their antagonistic activities using representative pathogenic bacteria harboring antibiotic resistance for final selection. Growth inhibitory activities against two representative indicator strains (*E*. *coli* CCARM 1G440 and *S*. *aureus* CCARM 3A860) were tested. In all cases, the diameters of inhibition zones were >12 mm (Table 2). Among the five strains selected, NSMJ23 showed the highest inhibition values against the two indicator strains.

#### 3.1.2. Phylogenetic Affiliation of the Five Potential LAB Probiotic Strains

**Figure 1:**
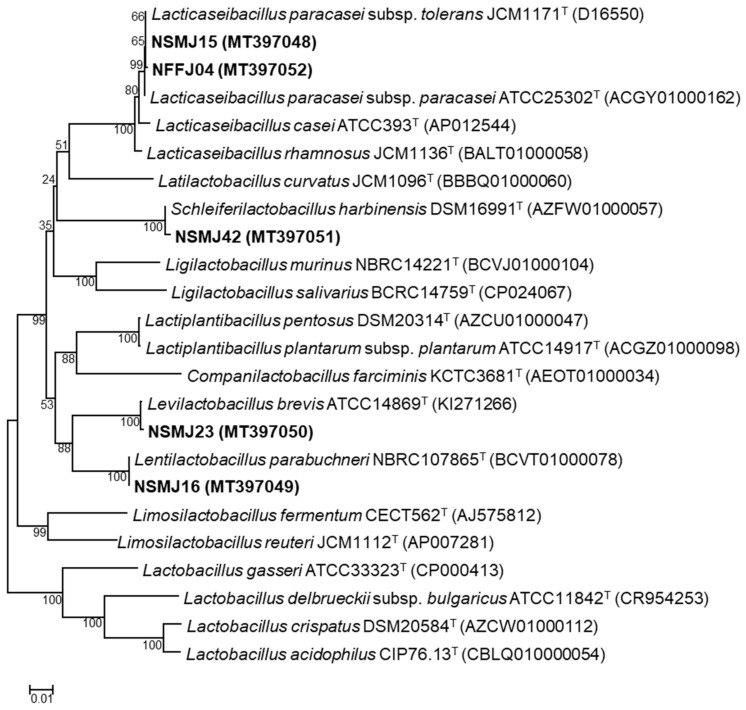
Phylogenetic analysis of five selected lactic acid bacteria (LAB) strains based on their 16S rRNA gene sequences. *Bacillus subtilis* subsp. *subtilis* NCIB3610^T^ was used as an outgroup (not shown). The scale bar indicates the number of changes per nucleotide position.

Phylogenetic affiliation based on 16S rRNA genes of the five strains showing good acid and bile salt tolerance, cell surface hydrophobicity, adhesion to Caco-2 cells, and antagonistic activities revealed that these five strains exhibited very high sequence similarities of 99.58–100% to the type strains of species (Table S1). Two strains, NSMJ15 and NFFJ04, shared 100 and 99.93% sequence similarities with 16S rRNA sequences of *Lacticaseibacillus paracasei* (formerly *Lactobacillus paracasei*), respectively. The other, three strains, NSMJ16, NSMJ23, and NSMJ42, were closely related to *Lentilactobacillus parabuchneri* (formerly *Lactobacillus parabuchneri*), *Levilactobacillus brevis* (formerly *Lactobacillus brevis*), and *Schleiferilactobacillus harbinensis* (formerly *Lactobacillus harbinensis*), sharing 100, 99.87, and 99.58% sequence similarities, respectively. BLAST searches and phylogenetic tree analysis showed that these five LAB strains belong to *Lacticaseibacillus*, *Lentilactobacillus*, and *Schleiferilactobacillus* (Figure 1).

#### 3.1.3. Phenotypic Characterization of the Five Potential Probiotic LAB Strains

The capability of the five LAB strains to metabolize 49 carbohydrate substrates was evaluated through the API 50 CH assay. Fermentation profiles of carbohydrates for the five strains were highly variable (Figure S3). C6 hexoses (D-galactose, D-glucose, and D-fructose), disaccharide (D-maltose), and salts (potassium gluconate) were carbohydrate substrates that could be metabolized by all five strains. In particular, in terms of symbiotic utilization [39], carbohydrate fermentation profiling showed that inulin could be utilized by strain NFFJ04 and D-raffinose could be utilized by strains NSMJ16 and NFFJ04. The growth properties of the five strains were tested. Results demonstrated that they could grow under intestinal tract conditions (Table S2). For hemolysis, none of these strains was able to hydrolyze sheep blood, proving their non-hemolytic activity (data not shown).

### 3.2. In-Vitro Characterization of Growth Inhibitory Activity against the Gut Pathogen of Selected Five LAB Strains Using Microbiota Composition Analysis

#### 3.2.1. Change in Growth Kinetics of the Fecal Microbiome by CFS Treatment

A growth kinetics analysis of the fecal microbiome in CFS-treatment samples revealed that NSMJ15- and NSMJ42-CFS have a growth-inhibiting effect on fecal microorganisms (Figure S4). In the cases of NSMJ16-, NSMJ23-, and NFFJ04-CFS, there was no significant difference of growth kinetics compared to the non-treatment (control) sample.

#### 3.2.2. Analysis of Bacterial Diversity and Community Composition after CFS Treatment

To investigate in detail the change in bacterial community composition by CFS treatment, Illumina-based 16S amplicon sequencing was performed. A total of 1,327,731 bacterial 16S rRNA gene sequencing reads were generated using the Illumina MiSeq platform. After removing low-quality reads, including chimeric sequences, 429,539 reads (32.4% of total sequencing reads) were used to analyze microbial compositions in fecal samples. Evaluation of bacterial diversity changes in control (non-treated) and CFS-treated samples during in vitro cultivation in batch culture showed the bacterial diversities decreased for all five CFS-treated samples compared to the control after 8 and 16 h incubation. Statistical diversity indices, such as Shannon–Weaver and Inverse Simpson indices, supported these results of bacterial diversity changes (Table 3).

**Figure 2:**
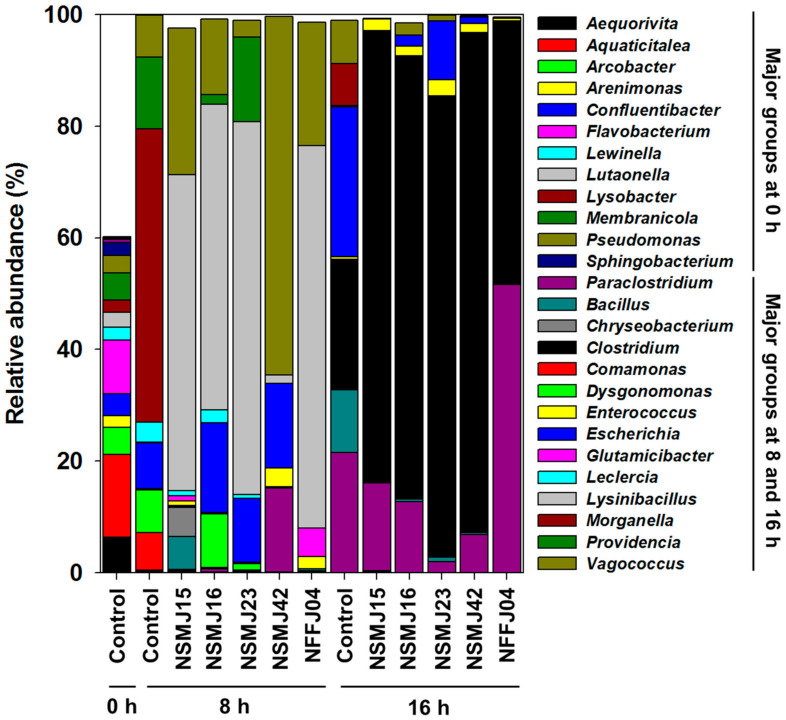
Changes in relative abundance (%) of bacterial taxonomic groups in controls (no treatment) and five CFS-treated samples at the genus level. Genera composed of taxonomic compositions above 2.0% of the total reads in all respective samples are shown.

**Figure 3:**
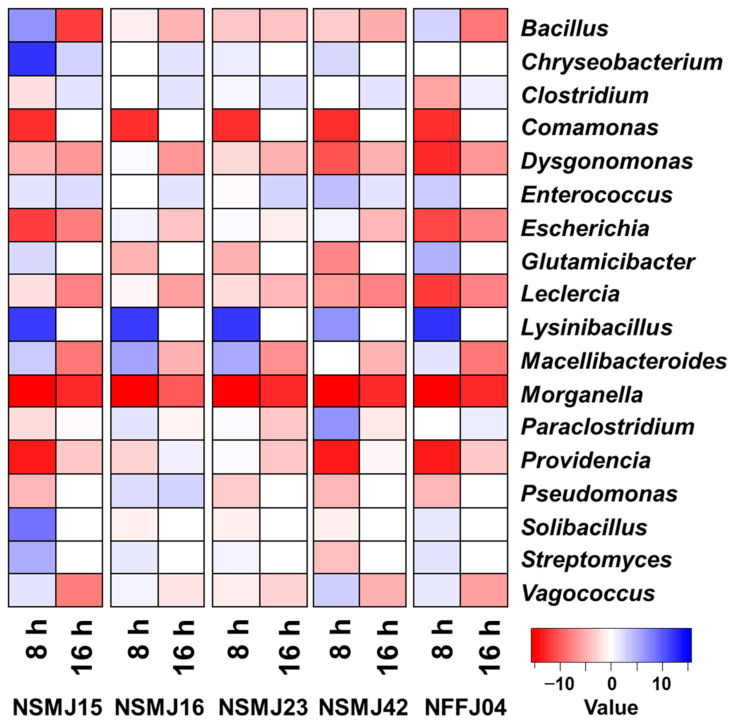
Heat map showing log2-fold change relative to controls at the genus level. Red and blue cells indicate decreased and increased abundance due to treatment, respectively.

Sequencing reads were classified at genus levels to detect changes of the bacterial population in samples during incubation after CFS treatment (Figure 2 and Figure 3). In the control fecal sample (0 h) of the test subject, the phyla *Bacteroidetes* (62.4%), *Proteobacteria* (25.1%), *Actinobacteria* (2.4%), and *Firmicutes* (2.4%) predominated. At the genus level, *Aquaticitalea* (14.8%), *Flavobacterium* (9.6%), *Aequorivita* (6.4%), *Membranicola* (4.9%), *Arcobacter* (4.8%), *Confluentibacter* (4.1%), and *Pseudomonas* (3.1%) were detected as major microbial community members (Figure 2 and Figure S5). After 8 h of anaerobic incubation of fecal microbiota in BHI culture media, there was a shift in the major bacterial populations. *Morganella* (52.5%), *Providencia* (12.8%), *Escherichia* (8.2%), *Dysgonomonas* (7.7%), *Vagococcus* (7.6%), *Comamonas* (6.7%), and *Leclercia* (3.6%) were detected as major bacterial genera, followed by *Enterococcus* (0.2%), *Clostridium* (0.2%), *Glutamicibacter* (0.2%), and *Paraclostridium* (0.1%) (Figure 2). After 16 h of incubation, *Escherichia* (26.9%), *Clostridium* (23.3%), *Paraclostridium* (21.5%), *Bacillus* (11.2%), *Vagococcus* (7.7%), *Morganella* (7.5%), *Enterococcus* (0.4%), and *Macellibacteroides* (0.3%) predominated, followed by *Leclercia* (0.2%) and *Dysgonomonas* (0.1%) (Figure 2). Among the major genera detected during 16 h of incubation, *Escherichia*, *Clostridium*, *Bacillus*, *Vagococcus*, *Morganella*, *Enterococcus*, *Leclercia*, and *Providencia* are known as undesirable bacterial groups, including (emerging) pathogens. Accordingly, the bacterial community analysis from control to CFS-treatment samples enabled the assessment of antimicrobial abilities against gut pathogens for strains NSMJ15, NSMJ16, NSMJ23, NSMJ42, and NFFJ04.

In all five CFS-treated samples, the bacterial community change occurred markedly compared to the control at both times (Figure 2 and Figure 3). Although the impact of CFS on bacterial community composition in fecal samples was generally similar between all five strains, there was little variability in the increase or decrease of bacterial members and/or the change rate (Figure 2 and Figure 3). Both *Morganella* and *Leclercia* were decreased in all samples at both times. *Comamonas* and *Lysinibacillus*, which were not detected at 16 h of incubation, were decreased in all samples at 8 h. *Bacillus*, *Vagococcus*, and *Escherichia* were decreased in all samples at 16 h. On the other hand, *Clostridium* was increased in all samples at 16 h. In the case of the NSMJ15 CFS-treated sample, *Moganella*, *Providencia*, *Escherichia*, *Dysgonomonas*, *Comamonas*, and *Leclercia* were decreased by 52.5, 12.8, 8.2, 7.4, 6.7, and 2.8%, respectively, with increases of *Lynsinibacillus*, *Vagococcus*, *Bacillus,* and *Chryseobacterium* at 8 h of incubation. *Escherichia*, *Bacillus*, *Vagococcus*, *Moganella*, and *Paraclostridium* were decreased by 26.8, 11.2, 7.7, 7.5, and 5.8%, respectively, with increases of *Clostridium* and *Enterococcus* at 16 h of incubation. In the case of the NSMJ16 CFS-treated sample, *Moganella*, *Providencia*, *Comamonas*, and *Leclercia* were decreased by 52.5, 11.0, 6.7, and 1.2%, respectively, with increases of *Lynsinibacillus*, *Escherichia*, *Vagococcus,* and *Dysgonomonas* at 8 h. *Escherichia*, *Bacillus*, *Paraclostridium*, *Moganella*, and *Vagococcus* were decreased 24.9, 10.8, 8.8, 7.5, and 5.5%, respectively, with increases of *Clostridium* and *Enterococcus* at 16 h. In the case of the NSMJ23 CFS-treated sample, *Moganella*, *Comamonas*, *Dysgonomonas*, *Vagococcus*, and *Leclercia* were decreased by 52.5, 6.7, 6.5, 4.6, and 2.9%, respectively, with increases of *Lynsinibacillus*, *Escherichia*, and *Providencia* at 8 h. *Paraclostridium*, *Escherichia*, *Bacillus*, *Morganella*, and *Vagococcus* were decreased by 19.6, 16.4, 10.4, 7.5, and 6.7%, respectively, with increases of *Clostridium* and *Enterococcus* at 16 h. In the case of the NSMJ42 CFS-treated sample, *Moganella*, *Providencia*, *Dysgonomonas*, *Comamonas*, and *Leclercia* were decreased by 52.5, 12.8, 7.7, 6.7, and 3.5%, respectively, with increases of *Vagococcus*, *Paraclostridium*, *Escherichia*, *Enterococcus*, and *Lynsinibacillus* at 8 h. *Escherichia*, *Paraclostridium*, *Bacillus*, *Morganella*, and *Vagococcus* were decreased by 25.7, 14.7, 10.9, 7.5, and 7.5%, respectively, with increases of *Clostridium* and *Enterococcus* at 16 h. In the case of the NFFJ04 CFS-treated sample, *Morganella*, *Providencia*, *Escherichia*, *Dysgonomonas*, *Comamonas*, and *Leclercia* were decreased by 52.5, 12.8, 8.2, 7.7, 6.7, and 3.6%, respectively, with increases of *Lynsinibacillus*, *Vagococcus*, *Glutamicibacter*, and *Enterococcus* at 8 h. *Escherichia*, *Bacillus*, *Vagococcus*, and *Morganella* were decreased by 26.7, 11.2, 7.6, and 7.5%, respectively, with increases of *Paraclostridium* and *Clostridium* at 16 h. Along with these results, a heatmap showing log2-fold change relative to controls clearly showed increases or decreases of bacterial groups at both times after CFS treatment (Figure 3).

The results of species-level classification showed that most of the genera were represented by few species (Figure 2, Figure 3, and Figure S6). Interestingly, OTUs belonging to the major genera *Bacillus*, *Escherichia*, *Leclercia, Morganella*, and *Vagococcus*, which were decreased in all CFS-treated samples as time passed, were mostly classified as pathogenic species—*Bacillus wiedmanii*, *Escherichia fergusonii*, *Leclercia adecarboxylate, Morganella morganii*, and *Vagococcus fluvialis* (Figure S6). The members of *Vibrio* (0.23% at 16 h of control) detected as rare populations at the genus-level were also decreased in all CFS samples (data not shown), the OTUs of which were classified as belonging to *Vibrio vulnificus* (Figure S6). The OTUs of *Clostridium* that increased in all CFS samples were mostly classified to *Clostidium butyricum*.

## 4. Discussion

To have probiotic effects in the intestinal tract, probiotic microbes must be able to survive in the gastrointestinal tract environment [23,40]. Thus, resistance to the low pH (pH 1.5–3.0) of the stomach is one of the most important selection criteria for probiotics [41]. Among the 54 bacterial isolates, for 12 strains belonging to *Leuconostoc mesenteroides*, *Latilactobacillus curvatus*, *Lacticaseibacillus paracasei*, *Lentilactobacillus parabuchneri*, *Levilactobacillus brevis*, and *Schleiferilactobacillus harbinensis*, survival at pH 2.5 was above 60%. These values were similar to that of the LGG strain (Figure S1A). The ability of these LAB strains to survive at pH 2.5 has been reported previously [42,43,44]. A microorganism is considered to have a good tolerance if it is resistant to 0.3% (*v*/*v*) of bile salt [45,46,47]. The concentration was selected to mimic the physiological conditions and is often used when screening potential probiotics for their bile tolerance [47]. Of the 12 primary selected LABs, survival rates at 0.3% bile salt for eight of the LABs, excluding the strains NKJ96, NKJ198, NKJ235 and NSMJ56, were close to that of the LGG strain (Figure S1B). Another important criterion to select a candidate for probiotic use is its ability to adhere to intestinal mucosal cells, which is a pre-requisite for longer permanence in the digestive tract, as well as functionalities such as control of harmful microbes and modulation of the immune system [48]. In addition, cell-surface hydrophobicity is a consideration which appears to help in bacterial adhesion [48,49]. By combining in vitro determination of bacterial adhesion to Caco-2 cell and hydrophobicity (Figure S2), five LAB strains (*Lacticaseibacillus paracasei* strains NSMJ15 and NFFJ04, *Lentilactobacillus parabuchneri* NSMJ16, *Levilactobacillus brevis* NSMJ23, and *Schleiferilactobacillus harbinensis* NSMJ42) were selected for showing inhibitory abilities against pathogenic bacteria. Bacterial infection in the gastrointestinal (GI) tract is a major cause of disease in humans and animals which can severely threaten individuals and has a global impact on human health and on animal industries [50]. *Escherichia coli*, *Klebsiella*, *Salmonella*, *Shigella*, *Staphylococcus*, and *Campylobacter* spp. are the most-reported (opportunistic) pathogens. They can cause diseases such as inflammation, diarrhea, fever, abdominal cramps, and vomiting in human and animals. Besides those just named, other kinds of microbe are being continuously reported with similar pathogenicity profiles. Antimicrobial resistance (AMR) genes are thus of great concern to public health and to animal industries [51,52,53].

Given the seriousness of the threat posed by antibiotic-resistant pathogens to public health [54], a number of research groups involved in antibiotic development have shifted towards alternative therapies to treat the infections they cause. These strategies are divided into three main categories: (1) naturally occurring alternatives, including phage therapy [55], antimicrobial peptides [56], bacteriocins [57], and antibodies [58]; (2) synthetically designed strategies, such as synthetic mimics of antimicrobial peptides (SMAMPs) [59] and antibacterial oligonucleotides [60]; and (3) biotechnology-based approaches, such as genetically modified phages [61], lysins [62], and antibiotic inactivators [63]. These approaches have suggested promising alternatives to conventional antibiotic treatments. However, most of these alternative approaches are strain- or species-specific, as opposed to the broad-spectrum activity of conventional antibiotics. Probiotics can also be considered as an alternative to antibiotics. They are being applied in the treatment of various gastrointestinal infections [64,65,66]. In this study, all five LAB strains showed growth inhibition against *Staphylococcus aureus* and *Escherichia coli*, which are representatives of Gram-positive and -negative pathogenic bacteria and have antibiotic resistance (Table 2). And through in vitro batch-culture of fecal microbiomes and 16S rRNA amplicon sequencing, we found that the genera containing pathogenic bacteria species (*Morganella*, *Leclercia, Bacillus*, *Escherichia*, *Vagococcus*, and *Vibrio*) were reduced in all five CFS-treated samples (Figure 2, Figure 3, and Figure S6). The 16S rRNA gene amplicon sequencing using variable regions may not be suitable for high-resolution gut microbiota profiling at the species-level [67,68]. Nevertheless, along with the genus-level classifications (Figure 2 and Figure 3), the species-level classifications from control to CFS-treatment samples supported the antimicrobial potential of five LAB strains (Figure S6). OTUs belonging to the above genera were mostly classified as *Bacillus wiedmanii*, *Escherichia fergusonii*, *Leclercia adecarboxylata*, *Morganella morganii*, *Vagococcus fluvialis*, and *Vibrio vulnificus* (Figure S6). These species were regarded as pathogens that could be responsible for human or animal diseases and their extended-spectrum resistance to antibiotics has been reported [69,70,71,72,73,74,75,76,77,78,79]. Despite their similar impact in terms of compositional change with respect to pathogens, strains NSMJ15 and NSMJ42 might be highly effective for growth inhibition against gut pathogens in the gut (Figure S4). These data suggest that these two strains secrete inhibitory factors that could suppress the entire microbiome, not just pathogens. LABs generally produce several inhibitory factors, such as metabolic end-products, e.g., bacteriocins, numerous organic acids, and hydrogen peroxides [80]. The types of inhibitory factors and their activities are diverse. In order to find clues as to these inhibitory properties, studies looking to find inhibitory factors and identify them through comparative analysis of the five LAB strains should be commenced.

Overall, our results showed that the five LAB strains screened in our study could regulate the growth of gut pathogens harboring antibiotic resistance in the gut. Although this study did not reveal which processes could control the pathogens, it is assumed that the pathogens could be inhibited by LAB-originated organic acids and/or antimicrobial factors in CFS or by manipulating competition with their neighbors for available nutrients within microbial communities. There is a need to analyze the genome of these LAB strains to determine the mechanisms of microbial antagonism.

## 5. Conclusions

In this study, *Lacticaseibacillus paracasei* NSMJ15, *Lentilactobacillus parabuchneri* NSMJ16, *Levilactobacillus brevis* NSMJ23, *Schleiferilactobacillus harbinensis* NSMJ42, and *Lacticaseibacillus paracasei* NFFJ04 were newly isolated as potentially probiotic candidates through in vitro study. In particular, the reduction of the members of *Bacillus*, *Escherichia*, *Leclercia*, *Morganella*, *Vagococcus*, and *Vibrio* spp. in their culture supernatants suggested that these strains have antimicrobial abilities against gut pathogens. Although these results were obtained from an in vitro batch culture system, it appears that these LAB strains would exert microbial modulation effects in terms of pathogen control in the gut. Their probiotic characteristics could be of great interest as novel probiotic candidates and biotherapeutic agents, or as supplements for human or animal health. Further genomic studies and in vivo studies examining the role of these LABs in the host-gut microbiome are needed in the future.

## Figures and Tables

**Table 1:** Acid and bile tolerance, adhesion (%) to Caco-2 cells, and hydrophobicity (%) of five selected lactic acid bacteria (LAB) strains.

Strain	Acid Tolerance			Bile Salt Tolerance			Adhesion to Caco-2 Cell (%)	Hydrophobicity (%)
	Control (log CFU/mL)	pH 2.5 (log CFU/mL)	Survival Rate (%)	Control (log CFU/mL)	0.3% (*w*/*v*) BS (log CFU/mL)	Survival Rate (%)		
NSMJ15	8.65 ± 0.50	6.35 ± 1.17	73.11	9.96 ± 0.15	8.89 ± 0.10	89.23	14.93 ± 1.54	88.20 ± 7.18
NSMJ16	8.58 ± 0.66	7.02 ± 0.68	81.77	9.58 ± 0.17	8.99 ± 0.14	93.82	8.08 ± 0.95	80.64 ± 6.16
NSMJ23	8.16 ± 0.59	6.21 ± 0.65	76.08	9.61 ± 0.02	9.36 ± 0.08	97.52	26.19 ± 5.56	78.19 ± 13.81
NSMJ42	9.97 ± 0.25	8.06 ± 0.25	80.86	10.26 ± 0.27	9.55 ± 0.08	93.29	26.73 ± 4.68	78.32 ± 5.20
NFFJ04	9.79 ± 0.06	6.86 ± 0.52	70.02	10.24 ± 0.04	9.61 ± 0.01	93.97	7.62 ± 0.61	57.12 ± 3.23
LGG	9.79 ± 0.20	7.00 ± 0.67	71.60	10.11 ± 0.22	9.03 ± 0.24	89.32	19.50 ± 7.82	26.39 ± 5.01

Each value represents the mean value ± standard deviation (SD) from triplicate experiments.

**Table 2:** Assays of antagonistic activities of five selected lactic acid bacteria (LAB) strains.

Strains	Antagonistic Activity ^a^	
	*E*. *coli* CCARM 1G440	*S. aureus* CCARM 3A860
NSMJ15	+++	+
NSMJ16	+++++	++++
NSMJ23	+++++	+++++
NSMJ42	+++	++++
NFFJ04	+++	+++

^a^ Diameter (mm) of inhibition zones around colonies showing antimicrobial activities. +, 12–15; +++, 18–21; ++++, 21–24; +++++, 24–27.

**Table 3:** Summary and statistical bacterial diversities of Illumina sequencing data obtained from controls (non-treated) and cell free supernatant (CFS)-treated samples.

Samples		No. of Reads	No. of High-Quality Reads	Average Read Length (bp)	OTUs ^a^	Chao1 ^a^	Shannon ^a^	Inverse Simpson ^a^
0 h	Non-treated	100,495	11,623	457	332	359.51	6.13	0.96
8 h	Non-treated	113,395	43,322	464	64	94.00	2.34	0.69
	NSMJ15	113,312	38,697	464	123	176.81	1.93	0.60
	NSMJ16	97,465	36,225	464	60	79.46	2.03	0.65
	NSMJ23	107,232	42,360	465	66	85.50	1.60	0.52
	NSMJ42	86,614	33,807	460	83	131.75	1.58	0.54
	NFFJ04	87,505	28,044	463	75	134.50	1.41	0.48
16 h	Non-treated	104,636	32,494	452	67	106.55	2.59	0.80
	NSMJ15	89,975	27,654	441	79	98.12	1.05	0.34
	NSMJ16	102,863	29,971	441	55	132.50	1.21	0.36
	NSMJ23	109,505	36,047	443	46	59.33	1.05	0.32
	NSMJ42	113,993	37,162	441	69	92.63	0.69	0.20
	NFFJ04	100,741	32,133	440	38	57.43	1.23	0.52

Abbreviation: OTUs, operational taxonomic units. ^a^ Diversity indices were calculated from the QIIME pipeline based on 16S rRNA gene sequencing reads.

## Data Availability

Illumina 16S amplicon sequencing data from this study are publicly available in the NCBI Short Read Archive (SRA) with accession number PRJNA762615. The accession numbers of 16S rRNA gene sequences are presented in Table S1.

## Supplementary Materials

The following are available online at https://www.mdpi.com/article/10.3390/microorganisms9102141/s1, Figure S1: (A) Acid and (B) bile salt tolerance (%) of the 12 primarily selected LAB strains. Figure S2: Cell surface hydrophobicity (%, MATS) and bacterial adhesion to Caco-2 cell (%) of the 12 primarily selected LAB strains. Figure S3: Fermentative profiling of five selected LAB strains from the API 50 CHL system assay. Figure S4: Growth kinetic curves of fecal microbiome treated with cell-free culture supernatant (CFS) from the five LAB strains NSMJ15, NSMJ16, NSMJ23, NSMJ42, and NFFJ04. Data points represent the means of triplicate replications and error bars represent standard deviations of means. Figure S5: Abundance profiling of bacterial communities in original sample (0 h) at the (A) phylum and (B) genus level. Others in B are composed of taxonomic compositions showing less than 0.1% of total reads in genus-level analyses. Figure S6: (A) Change in relative abundance (%) of bacterial taxonomic groups in the control and five CFS-treated samples at the species-level at 8 and 16 h of incubation. Species composed of taxonomic compositions above 0.2% of the total reads in all respective samples are shown. (B) Heat map showing log2-fold change relative to controls at the species level. Red and blue cells indicate decreased and increased abundance due to treatment, respectively. Table S1: Identification of primarily selected 12 LAB strains showing good acid tolerance (%) based on their 16S rRNA gene sequences. Table S2: Growth characteristics of the five selected LAB strains.

## References

[1] Belkaid Y., Hand T.W. (2014). Role of the microbiota in immunity and inflammation. Cell.

[2] Jandhyala S.M., Talukdar R., Subramanyam C., Vuyyuru H., Sasikala M., Nageshwar Reddy D. (2015). Role of the normal gut microbiota. World J. Gastroenterol..

[3] Ubeda C., Djukovic A., Isaac S. (2017). Roles of the intestinal microbiota in pathogen protection. Clin. Transl. Immunol..

[4] Bäumler A., Sperandio V. (2016). Interactions between the microbiota and pathogenic bacteria in the gut. Nature.

[5] Sekirov I., Russell S.L., Antunes L.C., Finlay B.B. (2010). Gut microbiota in health and disease. Physiol. Rev..

[6] Pickard J.M., Zeng M.Y., Caruso R., Núñez G. (2017). Gut microbiota: Role in pathogen colonization, immune responses, and inflammatory disease. Immunol. Rev..

[7] Niederwerder M.C. (2018). Fecal microbiota transplantation as a tool to treat and reduce susceptibility to disease in animals. Vet. Immunol. Immunopathol..

[8] Kim K.O., Gluck M. (2019). Fecal microbiota transplantation: An update on clinical practice. Clin. Endosc..

[9] Mulani M.S., Kamble E.E., Kumkar S.N., Tawre M.S., Pardesi K.R. (2019). Emerging strategies to combat ESKAPE pathogens in the era of antimicrobial resistance: A review. Front. Microbiol..

[10] Divya Ganeshan S., Hosseinidoust Z. (2019). Phage therapy with a focus on the human microbiota. Antibiotics.

[11] Rasmussen T.S., Koefoed A.K., Jakobsen R.R., Deng L., Castro-Mejía J.L., Brunse A., Neve H., Vogensen F.K., Nielsen D.S. (2020). Bacteriophage-mediated manipulation of the gut microbiome—Promises and presents limitations. FEMS Microbiol. Rev..

[12] Chaucheyras-Durand F., Durand H. (2010). Probiotics in animal nutrition and health. Benef. Microbes.

[13] Hemarajata P., Versalovic J. (2013). Effects of probiotics on gut microbiota: Mechanisms of intestinal immunomodulation and neuromodulation. Ther. Adv. Gastroenterol..

[14] Gao R., Zhang X., Huang L., Shen R., Qin H. (2019). Gut microbiota alteration after long-term consumption of probiotics in the elderly. Probiotics Antimicrob. Proteins.

[15] Liu W., Pang H., Zhang H., Cai Y., Zhang H., Cai Y. (2014). Biodiversity of Lactic Acid Bacteria. Lactic Acid Bacteria.

[16] Sánchez B., Delgado S., Blanco-Míguez A., Lourenço A., Gueimonde M., Margolles A. (2017). Probiotics, gut microbiota, and their influence on host health and disease. Mol. Nutr. Food Res..

[17] Azad M.A.K., Sarker M., Li T., Yin J. (2018). Probiotic species in the modulation of gut microbiota: An overview. Biomed. Res. Int..

[18] Markowiak P., Śliżewska K. (2018). The role of probiotics, prebiotics and synbiotics in animal nutrition. Gut Pathog..

[19] Hütt P., Shchepetova J., Lõivukene K., Kullisaar T., Mikelsaar M. (2006). Antagonistic activity of probiotic lactobacilli and bifidobacteria against entero- and uropathogens. J. Appl. Microbiol..

[20] Prabhurajeshwar C., Chandrakanth R.K. (2017). Probiotic potential of *Lactobacilli* with antagonistic activity against pathogenic strains: An in vitro validation for the production of inhibitory substances. Biomed. J..

[21] Li M., Wang Y., Cui H., Li Y., Sun Y., Qiu H.J. (2020). Characterization of lactic acid bacteria isolated from the gastrointestinal tract of a wild boar as potential probiotics. Front. Vet. Sci..

[22] Uyeno Y., Shigemori S., Shimosato T. (2015). Effect of probiotics/prebiotics on cattle health and productivity. Microbes Environ..

[23] García-Hernández Y., Pérez-Sánchez T., Boucourt R., Balcázar J.L., Nicoli J.R., Moreira-Silva J., Zoraya R., Héctor F., Odalys N., Albelo N. (2016). Isolation, characterization and evaluation of probiotic lactic acid bacteria for potential use in animal production. Res. Vet. Sci..

[24] Reuter J.A., Spacek D., Snyder M.P. (2015). High-throughput sequencing technologies. Mol. Cell.

[25] Wei X., Zhang Y., Zhou H., Tian F., Ni Y. (2019). Antimicrobial activities and in vitro properties of cold-adapted *Lactobacillus* strains isolated from the intestinal tract of cold water fishes of high latitude water areas in Xinjiang, China. BMC Microbiol..

[26] Walker D.K., Gilliland S.E. (1993). Relationships among bile tolerance, bile salt deconjugation, and assimilation of cholesterol by *Lactobacillus acidophilus*. J. Dairy Sci..

[27] Vinderola C.G., Reinheimer J.A. (2003). Lactic acid starter and probiotic bacteria: A comparative “in vitro” study of probiotic characteristics and biological barrier resistance. Food Res. Int..

[28] Cho Y.J., Kim D.H., Jeong D., Seo K.H., Jeong H.S., Lee H.G., Kim H. (2018). Characterization of yeasts isolated from kefir as a probiotic and its synergic interaction with the wine byproduct grape seed flour/extract. Food Sci. Technol..

[29] Bauernfeind A., Burrows J.R. (1978). Suggested procedure allowing use of plastic petri dishes in bacteriocin typing. Appl. Environ. Microbiol..

[30] Jeon H.H., Jung J.Y., Chun B.H., Kim M.D., Baek S.Y., Moon J.Y., Yeo S.H., Jeon C.O. (2016). Screening and characterization of potential *Bacillus* starter cultures for fermenting low-salt soybean paste (doenjang). J. Microbiol. Biotechnol..

[31] Yoon S.H., Ha S.M., Kwon S., Lim J., Kim Y., Seo H., Chun J. (2017). Introducing EzBioCloud: A taxonomically united database of 16S rRNA gene sequences and whole-genome assemblies. Int. J. Syst. Evol. Microbiol..

[32] Thompson J.D., Higgins D.G., Gibson T.J. (1994). CLUSTAL W: Improving the sensitivity of progressive multiple sequence alignment through sequence weighting, position specific gap penalties, and weight matrix choice. Nucleic Acids Res..

[33] Kumar S., Stecher G., Tamura K. (2016). MEGA7: Molecular Evolutionary Genetics Analysis Version 7.0 for Bigger Datasets. Mol. Biol. Evol..

[34] Magoč T., Salzberg S.L. (2011). FLASH: Fast length adjustment of short reads to improve genome assemblies. Bioinformatics.

[35] Li W., Fu L., Niu B., Wu S., Wooley J. (2012). Ultrafast clustering algorithms for metagenomic sequence analysis. Brief. Bioinform..

[36] Bolyen E., Rideout J.R., Dillon M.R., Bokulich N.A., Abnet C.C., Al-Ghalith G.A., Alexander H., Alm E.J., Arumugam M., Asnicar F. (2019). Reproducible, interactive, scalableand extensible microbiome data science using QIIME 2. Nat. Biotechnol..

[37] Zhang Z., Schwartz S., Wagner L., Miller W. (2000). A greedy algorithm for aligning DNA sequences. J. Comput. Biol..

[38] Warnes G.R., Bolker B., Bonebakker L., Gentleman R., Huber W., Liaw A., Lumley T., Maechler M., Magnusson A., Moeller S. (2020). gplots: Various R Programming Tools for Plotting Data. R Package Version 3.1.1. https://cran.r-project.org/web/packages/gplots/.

[39] Pandey K.R., Naik S.R., Vakil B.V. (2015). Probiotics, prebiotics and synbiotics—A review. J. Food Sci. Technol..

[40] Dunne C., O’Mahony L., Murphy L., Thornton G., Morrissey D., O’Halloran S., Feeney M., Flynn S., Fitzgerald G., Daly C. (2001). In vitro selection criteria for probiotic bacteria of human origin: Correlation with in vivo findings. Am. J. Clin. Nutr..

[41] Solieri L., Bianchi A., Mottolese G., Lemmetti F., Giudici P. (2014). Tailoring the probiotic potential of non-starter *Lactobacillus* strains from ripened Parmigiano Reggiano cheese by in vitro screening and principal component analysis. Food Microbiol..

[42] Chang J.H., Shim Y.Y., Cha S.K., Chee K.M. (2010). Probiotic characteristics of lactic acid bacteria isolated from kimchi. J. Appl. Microbiol..

[43] Lee H., Yoon H., Ji Y., Kim H., Park H., Lee J., Shin H., Holzapfel W. (2011). Functional properties of *Lactobacillus* strains isolated from kimchi. Int. J. Food Microbiol..

[44] Zhang B., Wang Y., Tan Z., Li Z., Jiao Z., Huang Q. (2016). Screening of probiotic activities of *Lactobacilli* strains isolated from traditional Tibetan Qula, a raw yak milk cheese. Asian-Australas. J. Anim. Sci..

[45] Gilliland S.E., Staley T.E., Bush L.J. (1984). Importance of bile tolerance of *Lactobacillus acidophilus* used as a dietary adjunct. J. Dairy Sci..

[46] Charteris W.P., Kelly P.M., Morelli L., Collins J.K. (1998). Development and application of an in vitro methodology to determine the transit tolerance of potentially probiotic *Lactobacillus* and *Bifidobacterium* species in the upper human gastrointestinal tract. J. Appl. Microbiol..

[47] Gotcheva V., Hristozova E., Hristozova T., Guo M., Roshkova Z., Angelov A. (2002). Assessment of potential probiotic properties of lactic acid bacteria and yeast strains. Food Biotechnol..

[48] Schillinger U., Guigas C., Holzapfel W.H. (2005). In vitro adherence and other properties of lactobacilli used in probiotic yoghurt-like products. Int. Dairy J..

[49] Krausova G., Hyrslova I., Hynstova I. (2019). In vitro evaluation of adhesion capacity, hydrophobicity, and auto-aggregation of newly isolated potential probiotic strains. Fermentation.

[50] Zhang Y.J., Li S., Gan R.Y., Zhou T., Xu D.P., Li H.B. (2015). Impacts of gut bacteria on human health and diseases. Int. J. Mol. Sci..

[51] Ferri M., Ranucci E., Romagnoli P., Giaccone V. (2017). Antimicrobial resistance: A global emerging threat to public health systems. Crit. Rev. Food Sci. Nutr..

[52] Bengtsson B., Greko C. (2014). Antibiotic resistance—Consequences for animal health, welfare, and food production. Upsala J. Med. Sci..

[53] Cerniglia C.E., Pineiro S.A., Kotarski S.F. (2016). An update discussion on the current assessment of the safety of veterinary antimicrobial drug residues in food with regard to their impact on the human intestinal microbiome. Drug Test. Anal..

[54] Ghosh C., Sarkar P., Issa R., Haldar J. (2019). Alternatives to Conventional Antibiotics in the Era of Antimicrobial Resistance. Trends Microbiol..

[55] Kutter E., De Vos D., Gvasalia G., Alavidze Z., Gogokhia L., Kuhl S., Abedon S.T. (2010). Phage therapy in clinical practice: Treatment of human infections. Curr. Pharm. Biotechnol..

[56] Mandal S.M., Roy A., Ghosh A.K., Hazra T.K., Basak A., Franco O.L. (2014). Challenges and future prospects of antibiotic therapy: From peptides to phages utilization. Front. Pharm..

[57] Cotter P.D., Ross R.P., Hill C. (2013). Bacteriocins—A viable alternative to antibiotics?. Nat. Rev. Microbiol..

[58] Bebbington C., Yarranton G. (2008). Antibodies for the treatment of bacterial infections: Current experience and future prospects. Curr. Opin. Biotechnol..

[59] Scott R.W., Tew G.N. (2017). Mimics of host defense proteins; strategies for translation to therapeutic applications. Curr. Top. Med. Chem..

[60] Ayhan D.H., Tamer Y.T., Akbar M., Bailey S.M., Wong M., Daly S.M., Greenberg D.E., Toprak E. (2016). Sequence-specific targeting of bacterial resistance genes increases antibiotic efficacy. PLoS Biol..

[61] Pires D.P., Cleto S., Sillankorva S., Azeredo J., Lu T.K. (2016). Genetically engineered phages: A review of advances over the last decade. Microbiol. Mol. Biol. Rev..

[62] Nelson D.C., Schmelcher M., Rodriguez-Rubio L., Klumpp J., Pritchard D.G., Dong S., Donovan D.M. (2012). Endolysins as antimicrobials. Adv. Virus Res..

[63] Kokai-Kun J.F., Roberts T., Coughlin O., Sicard E., Rufiange M., Fedorak R., Carter C., Adams M.H., Longstreth J., Whalen H. (2017). The oral β-lactamase SYN-004 (Ribaxamase) degrades ceftriaxone excreted into the intestine in phase 2a clinical studies. Antimicrob. Agents Chemother..

[64] Liévin-Le Moal V., Servin A.L. (2014). Anti-infective activities of *Lactobacillus* strains in the human intestinal microbiota: From probiotics to gastrointestinal anti-infectious biotherapeutic agents. Clin. Microbiol. Rev..

[65] Lewis B.B., Pamer E.G. (2017). Microbiota-based therapies for *Clostridium difficile* and antibiotic-resistant enteric infections. Annu. Rev. Microbiol..

[66] Vieco-Saiz N., Belguesmia Y., Raspoet R., Auclair E., Gancel F., Kempf I., Drider D. (2019). Benefits and inputs from lactic acid bacteria and their bacteriocins as alternatives to antibiotic growth promoters during food-animal production. Front. Microbiol..

[67] Matsuo Y., Komiya S., Yasumizu Y., Yasuoka Y., Mizushima K., Takagi T., Kryukov K., Fukuda A., Morimoto Y., Naito Y. (2021). Full-length 16S rRNA gene amplicon analysis of human gut microbiota using MinION™ nanopore sequencing confers species-level resolution. BMC Microbiol..

[68] Johnson J.S., Spakowicz D.J., Hong B.Y., Petersen L.M., Demkowicz P., Chen L., Leopold S.R., Hanson B.M., Agresta H.O., Gerstein M. (2019). Evaluation of 16S rRNA gene sequencing for species and strain-level microbiome analysis. Nat. Commun..

[69] Funke G., Hany A., Altwegg M. (1993). Isolation of *Escherichia fergusonii* from four different sites in a patient with pancreatic carcinoma and cholangiosepsis. J. Clin. Microbiol..

[70] Lai C.C., Cheng A., Huang Y.T., Chung K.P., Lee M.R., Liao C.H., Hsueh P.R. (2011). *Escherichia fergusonii* bacteremia in a diabetic patient with pancreatic cancer. J. Clin. Microbiol..

[71] Weiss A.T.A., Lübke-Becker A., Krenz M., van der Grinten E. (2011). Enteritis and septicemia in a horse associated with infection by *Escherichia fergusonii*. J. Equine Vet. Sci..

[72] Savini V., Catavitello C., Talia M., Manna A., Pompetti F., Favaro M., Fontana C., Febbo F., Balbinot A., Di Berardino F. (2008). Multidrug-resistant *Escherichia fergusonii*: A case of acute cystitis. J. Clin. Microbiol..

[73] Forgetta V., Rempel H., Malouin F., Vaillancourt R., Topp E., Dewar K., Diarra M.S. (2012). Pathogenic and multidrug-resistant *Escherichia fergusonii* from broiler chicken. Poult. Sci..

[74] Liu H., Zhu J., Hu Q., Rao X. (2016). *Morganella morganii*, a non-negligent opportunistic pathogen. Int. J. Infect. Dis.

[75] Jones M.K., Oliver J.D. (2009). *Vibrio vulnificus*: Disease and pathogenesis. Infect. Immun..

[76] Heng S.P., Letchumanan V., Deng C.Y., Ab Mutalib N.S., Khan T.M., Chuah L.H., Chan K.G., Goh B.H., Pusparajah P., Lee L.H. (2017). *Vibrio vulnificus*: An environmental and clinical burden. Front. Microbiol..

[77] Jadhav K.P., Pai P.G. (2019). A rare infective endocarditis caused by *Vagococcus fluvialis*. J. Cardiol. Cases.

[78] Zhao Y., Chen C., Gu H.J., Zhang J., Sun L. (2019). Characterization of the genome feature and toxic capacity of a *Bacillus wiedmannii* isolate from the hydrothermal field in Okinawa Trough. Front. Cell Infect. Microbiol..

[79] Spiegelhauer M.R., Andersen P.F., Frandsen T.H., Nordestgaard R.L.M., Andersen L.P. (2019). *Leclercia adecarboxylata*: A case report and literature review of 74 cases demonstrating its pathogenicity in immunocompromised patients. Infect. Dis..

[80] Timothy B., Iliyasu A.H., Anvikar A.R. (2021). Bacteriocins of lactic acid bacteria and their industrial application. Curr Top. Lact Acid Bact. Probiotics.

