# Supplementary material for: In-Vitro Characterization of Growth Inhibition against the Gut Pathogen of Potentially Probiotic Lactic Acid Bacteria Strains Isolated from Fermented Products
Source: Microorganisms. 2021 Oct 13;9(10):2141. doi: 10.3390/microorganisms9102141 (PMC8537437; doi:10.3390/microorganisms9102141)
Supplement: Supplementary file 1 [file microorganisms-09-02141-s001.zip › microorganisms-1400086-supplementary.pdf]

Supplementary Materials:

Table S1. Identification of primarily selected 12 LAB strains showing good acid tolerance (%) based on their 16S rRNA gene sequences.

Strains	Closest type strain	Sequence similarity (%)	GenBank accession No.
NKJ81	*Leuconostoc mesenteroides* subsp. *jonggajibkimchii* DRC1506^T	100	MZ971173
NKJ96	*Latilactobacillus curvatus* JCM1096^T	100	MZ971174
NKJ170	*Leuconostoc mesenteroides subsp. jonggajibkimchii* DRC1506^T	100	MZ971175
NKJ198	*Leuconostoc mesenteroides subsp. mesenteroides* ATCC8293^T	100	MZ971176
NKJ235	*Latilactobacillus curvatus* JCM1096^T	100	MZ971177
NSMJ15	*Lacticaseibacillus paracasei* subsp. *tolerans* JCM 1171^T	100	MT397048
NSMJ16	*Lentilactobacillus parabuchneri* NBRC 107865^T	100	MT397049
NSMJ23	*Levilactobacillus brevis* ATCC 14869^T	99.87	MT397050
NSMJ27	*Lacticaseibacillus paracasei subsp. paracasei* ATCC 25302^T	100	MZ971178
NSMJ42	*Schleiferilactobacillus harbinensis* DSM 16991^T	99.58	MT397051
NSMJ56	*Lacticaseibacillus paracasei subsp. tolerans* JCM 1171^T	100	MZ971179
NFFJ04	*Lacticaseibacillus paracasei* subsp. *tolerans* JCM 1171^T	99.93	MT397052

Table S2. Growth characteristics of five selected LAB strains.

Characteristic	NSMJ15	NSMJ16	NSMJ23	NSMJ42	NFFJ04
Growth at					
Temperature (optimum, ℃)	10-45 (30-40)	10-40 (30-40)	10-40 (30-40)	10-45 (30-40)	10-45 (30-35)
pH (optimum)	4.0-8.0 (6.0)	3.5-8.0 (6.0-7.0)	3.5-8.0 (6.0-6.5)	3.0-9.0 (5.5-7.0)	4.0-8.0 (6.0-7.0)
NaCl (optimum, %)	0-5 (0-1)	0-7 (0-3)	0-5 (0-2)	0-6 (0-2)	0-6 (0-1)
Anaerobic condition	+	+	+	+	+

Supplementary figure legends

Figure S1. (A) Acid- and (B) bile salt tolerance (%) of primarily selected 12 LAB strains.

Figure S2. Cell surface hydrophobicity (%, MATS) and bacterial adhesion to Caco-2 cells (%) of primarily selected 12 LAB strains.

Figure S3. Fermentative profiling of five selected LAB strains from API 50 CHL system assay.

Figure S4. Growth kinetic curves of fecal microbiome treated with cell-free culture supernatant (CFS) from five LAB strains NSMJ15, NSMJ16, NSMJ23, NSMJ42, and NFFJ04. Data points represent means of triplicate replications and error bars represent standard deviations of means.

Figure S5. Abundance profiling of bacterial communities in original sample (0 h) at the (A) phylum and (B) genus level. Others in B are composed of taxonomic compositions showing less than 0.1% of total reads in genus level analyses.

Figure S6. (A) Change in relative abundance (%) of bacterial taxonomic groups in control and five CFS-treated samples at the species-level at 8 and 16 h of incubation. Species composed of taxonomic compositions above 0.2% of the total reads in all respective samples are shown. (B) Heat map showing log2-fold change relative to controls at the species level. Red and blue cells indicate decreased and increased abundance due to treatment, respectively.

Figure S1.

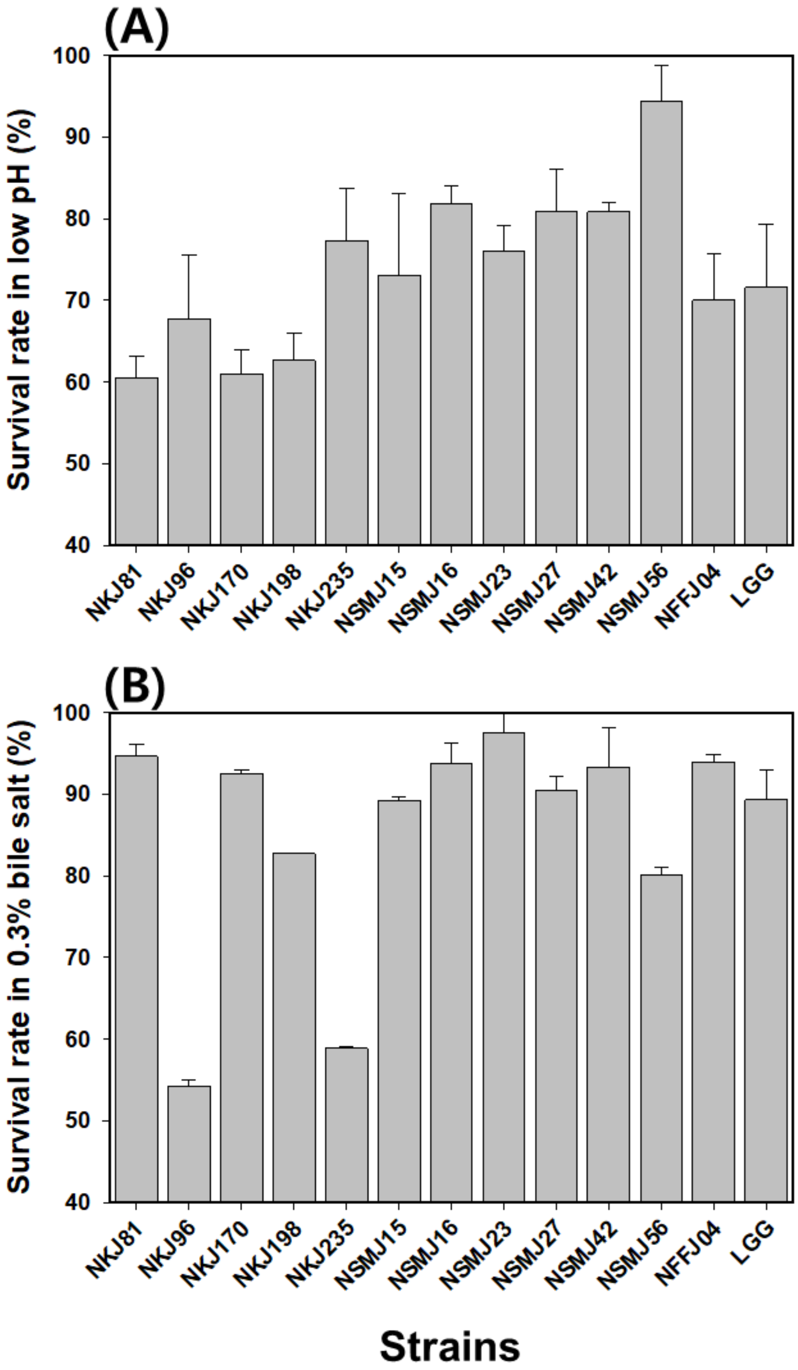
(A)
Survival rate in low pH (%)
100
90
80
70
60
50
40
NKJ81
NKJ96
NKJ170
NKJ198
NKJ235
NSMJ15
NSMJ16
NSMJ23
NSMJ27
NSMJ42
NSMJ56
NFFJ04
LGG
(B)
Survival rate in 0.3% bile salt (%)
100
90
80
70
60
50
40
NKJ81
NKJ96
NKJ170
NKJ198
NKJ235
NSMJ15
NSMJ16
NSMJ23
NSMJ27
NSMJ42
NSMJ56
NFFJ04
LGG
Strains

Figure S2.

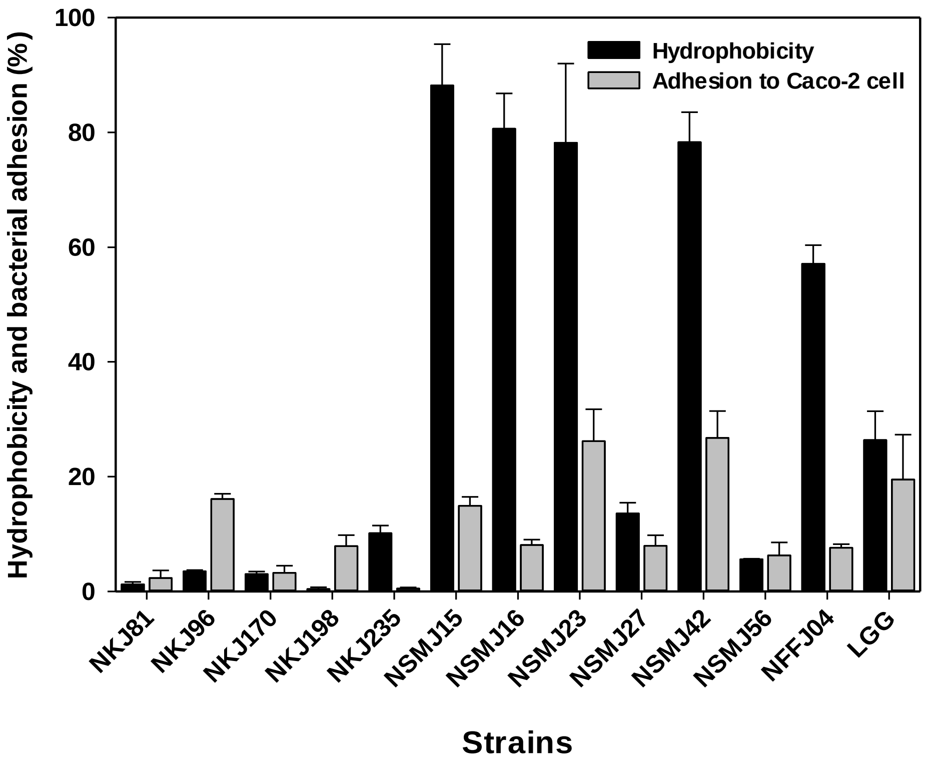

100
80
60
40
20
0
Hydrophobicity and bacterial adhesion (%)
Hydrophobicity
Adhesion to Caco-2 cell
NKJ81
NKJ96
NKJ170
NKJ198
NKJ235
NSMJ15
NSMJ16
NSMJ23
NSMJ27
NSMJ42
NSMJ56
NFFJ04
LGG
Strains

Figure S3.

No.	Utilization of	NSM J15	NSM J16	NSM J23	NSM J42	NFFJ 04	No.	Utilization of	NSM J15	NSM J16	NSM J23	NSM J27	NSM J42	NFFJ 04
0	Control						25	Esculin		■	■	■	■	■
1	Glycerol						26	Salicin				■	■	■
2	Erythritol						27	D-Cellibiose					■	■
3	D-arabinose						28	D-Maltose	■	■	■	■	■	■
4	L-arabinose		■	■	■		29	D-Lactose (bovine origin)				■		
5	D-ribose	■	■	■		■	30	D-Melibiose		■			■	
6	D-xylose			■	■		31	D-Saccharose (sucrose)		■		■	■	■
7	L-xylose						32	D-Trehalose	■			■	■	■
8	D-adonitol						33	Inulin						■
9	Methyl-ßD-xylopyranoside						34	D-Melezitose		■			■	■
10	D-galactose	■	■	■	■	■	35	D-Raffinose		■			■	
11	D-glucose	■	■	■	■	■	36	Amidon (starch)					■	
12	D-fructose	■	■	■	■	■	37	Glycogen						
13	D-mannose	■			■	■	38	Xylitol						
14	L-sorbose						39	Gentibiose					■	■
15	L-rhamnose						40	D-Turanose		■		■		■
16	Dulcitol	■					41	D-Lyxose						
17	Inositol						42	D-Tagatose	■			■	■	■
18	D-mannitol	■				■	43	D-Fucose						
19	D-sorbitol	■					44	L-Fucose						
20	Methyl-αD-mannopyranoside						45	D-arabitol						
21	Methyl-αD-glucopyranoside	■	■	■	■		46	L-arabitol						
22	N-acetylglucosamine	■		■	■	■	47	Potassium gluconate	■	■	■	■	■	■
23	Amygdalin				■	■	48	Potassium 2 ketogluconate			■			
24	Arbutin				■	■	49	Potassium 5 ketogluconate		■	■			

Figure S4.

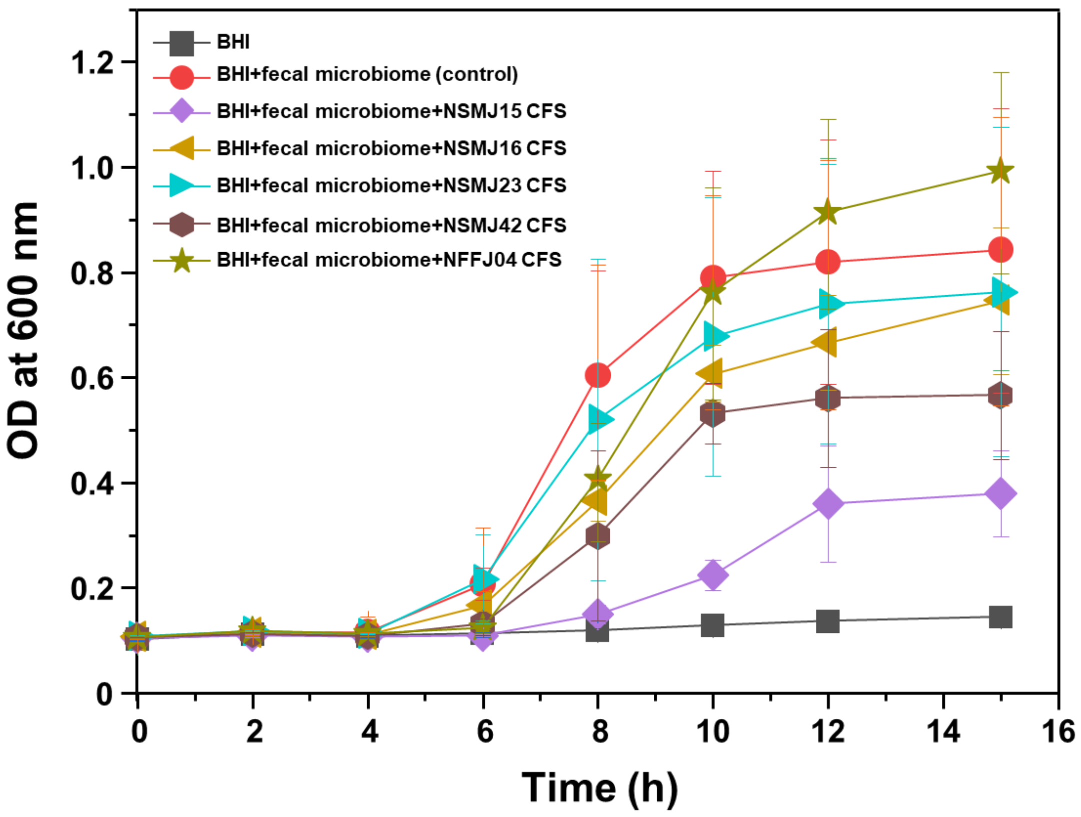

OD at 600 nm
1.2
1.0
0.8
0.6
0.4
0.2
0
0
2
4
6
8
10
12
14
16
Time (h)
BHI
BHI+fecal microbiome (control)
BHI+fecal microbiome+NSMJ15 CFS
BHI+fecal microbiome+NSMJ16 CFS
BHI+fecal microbiome+NSMJ23 CFS
BHI+fecal microbiome+NSMJ42 CFS
BHI+fecal microbiome+NFFJ04 CFS

Figure S5.

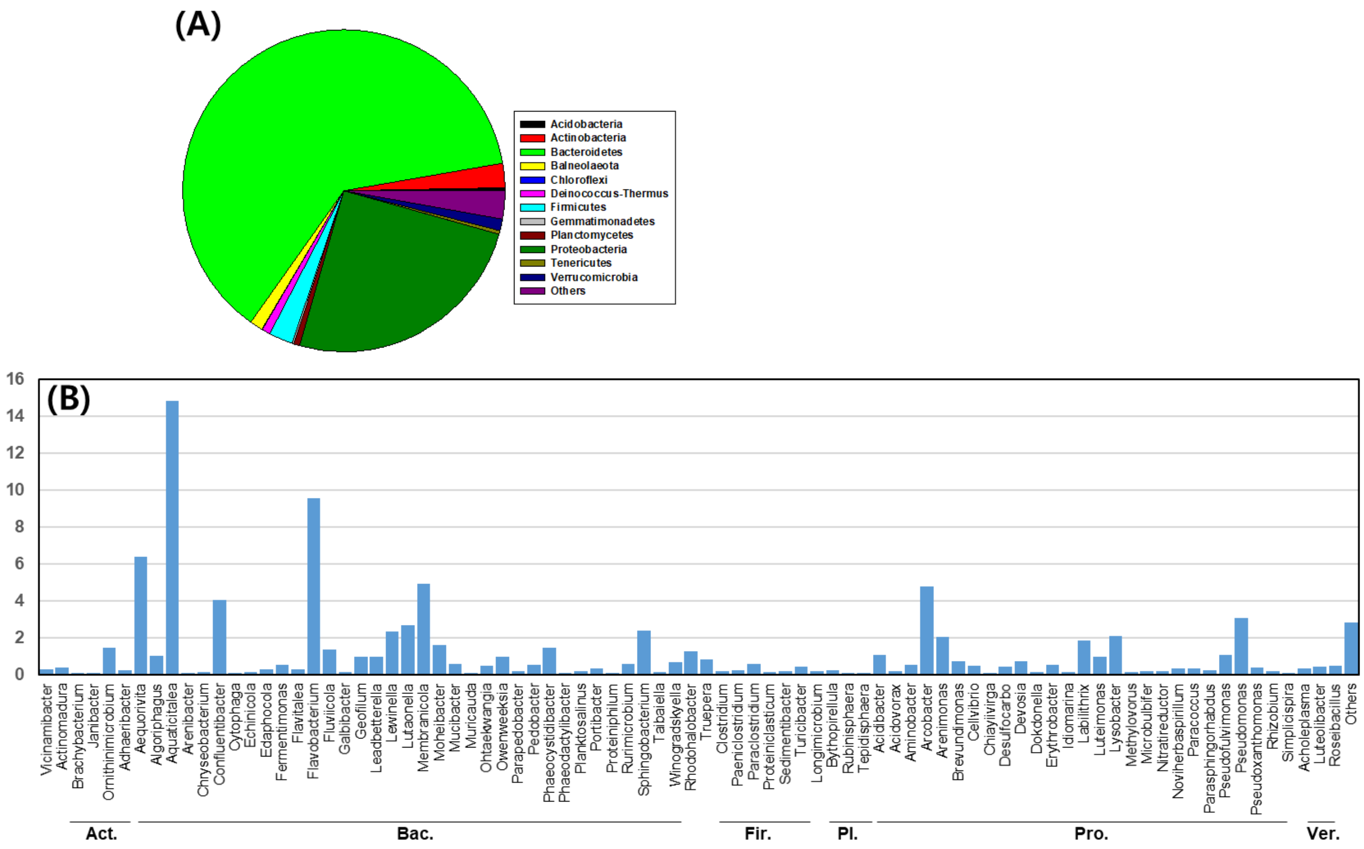
(A)
Acidobacteria
Actinobacteria
Bacteroidetes
Balneolaeota
Chloroflexi
Deinococcus-Thermus
Firmicutes
Gemmatimonadetes
Planctomycetes
Proteobacteria
Tenericutes
Verrucomicrobia
Others
(B)
16
14
12
10
8
6
4
2
0
Vicinamibacter
Actinomadura
Brachybacterium
Janibacter
Ornithinimicrobium
Adhaeribacter
Aequorivita
Algoriphagus
Aquaticitalea
Arenibacter
Chryseobacterium
Confluentibacter
Cytophaga
Echinicola
Edaphocola
Fermentimonas
Flavitalea
Flavobacterium
Fluviicola
Galbibacter
Geofilum
Leadbetterella
Lewinella
Lutaonella
Membranicola
Moheibacter
Mucibacter
Muricauda
Ohtaekwangia
Owenweeksia
Parapedobacter
Pedobacter
Phaeocystidibacter
Phaeodactylibacter
Planktosalinus
Portibacter
Proteiniphilum
Rurimicrobium
Sphingobacterium
Taibaiella
Winogradskyella
Rhodohalobacter
Truepera
Clostridium
Paeniclostridium
Paraclostridium
Proteiniclasticum
Sedimentibacter
Turicibacter
Longimicrobium
Bythopirellula
Rubinisphaera
Tepidisphaera
Acidibacter
Acidovorax
Aminobacter
Arcobacter
Arenimonas
Brevundimonas
Cellvibrio
Chiayiivirga
Desulfocarbo
Devosia
Dokdonella
Erythrobacter
Idiomarina
Labilithrix
Luteimonas
Lysobacter
Methylovorus
Microbulbifer
Nitratireductor
Noviherbaspirillum
Paracoccus
Parasphingorhabdus
Pseudofulvimonas
Pseudomonas
Pseudoxanthomonas
Rhizobium
Simplicispira
Acholeplasma
Luteolibacter
Roseibacillus
Others
Act.
Bac.
Fir.
Pl.
Pro.
Ver.

Figure S6.

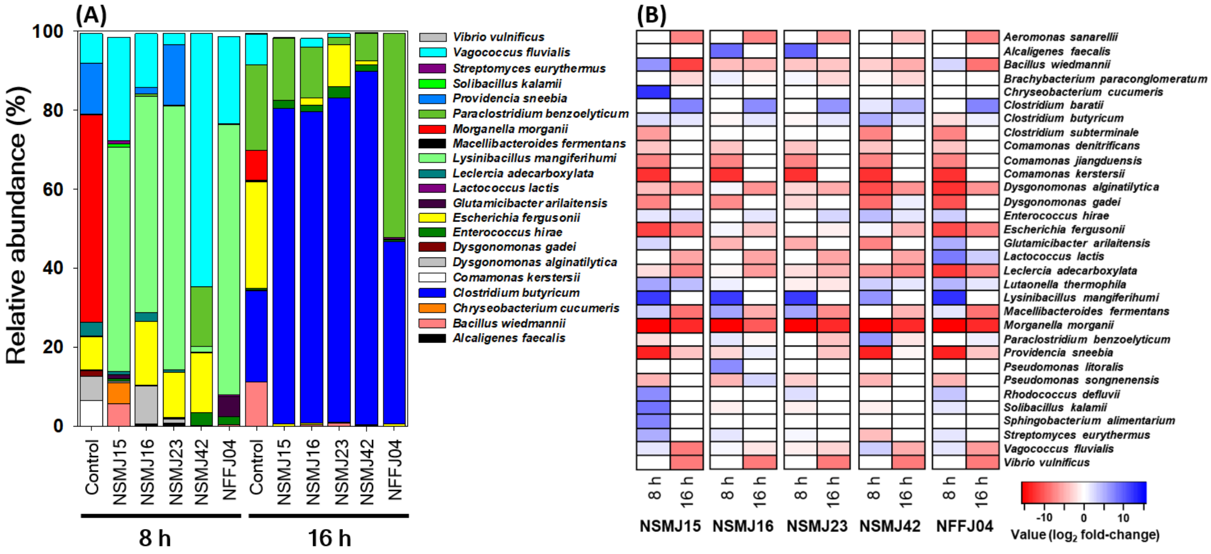

(A)
Relative abundance (%)
0
20
40
60
80
100
Control
NSMJ15
NSMJ16
NSMJ23
NSMJ42
NFFJ04
8 h
16 h
Vibrio vulnificus
Vagococcus fluvialis
Streptomyces eurythermus
Solibacillus kalamii
Providencia sneebia
Paraclostridium benzoelyticum
Morganella morganii
Macellibacteroides fermentans
Lysinibacillus mangiferihumi
Leclercia adecarboxylata
Lactococcus lactis
Glutamicibacter arilaitensis
Escherichia fergusonii
Enterococcus hirae
Dysgonomonas gadei
Dysgonomonas alginatilytica
Comamonas kerstersii
Clostridium butyricum
Chryseobacterium cucumeris
Bacillus wiedmannii
Alcaligenes faecalis
(B)
Aeromonas sanarellii
Alcaligenes faecalis
Bacillus wiedmannii
Brachybacterium paraconglomeratum
Chryseobacterium cucumeris
Clostridium baratii
Clostridium butyricum
Clostridium subterminale
Comamonas denitrificans
Comamonas jiangduensis
Comamonas kerstersii
Dysgonomonas alginatilytica
Dysgonomonas gadei
Enterococcus hirae
Escherichia fergusonii
Glutamicibacter arilaitensis
Lactococcus lactis
Leclercia adecarboxylata
Lutaonella thermophila
Lysinibacillus mangiferihumi
Macellibacteroides fermentans
Morganella morganii
Paraclostridium benzoelyticum
Providencia sneebia
Pseudomonas litoralis
Pseudomonas songnenensis
Rhodococcus defluvii
Solibacillus kalamii
Sphingobacterium alimentarium
Streptomyces eurythermus
Vagococcus fluvialis
Vibrio vulnificus
8 h
16 h
NSMJ15
NSMJ16
NSMJ23
NSMJ42
NFFJ04
-10
0
10
Value (log2 fold-change)
